# On the Influence of Compression by the Maternal Soft Parts during Natural Labor

**Published:** 1856-02

**Authors:** 


*On the Influence of Compression by the Maternal Soft Parts during Natural Labor.*—It occasionally happens in those accouchements where the employment of the forceps has been found necessary, that the infants born are attacked, immediately or a short time afterwards, by symptoms of congestion or compression of the brain. Fatal conseqnences frequently follow, and upon *post-mortem* examination, there are found considerable sanguineous effusions on the surface of the brain, injection of the choroid plexus, etc.; in short, lesions indicative of the injurious pressure to which the head had been exposed during labor.

M. Charrier details three such cases, the only difference being that the labor was natural and of ordinary duration; but, from the anatomical peculiarities presented by the infants, he considers both orders of cases as referable to the same immediate cause, viz: pressure on the brain.

In the first of these instances, the mother was healthy and the pelvis well formed; delivery was completed in six hours, and the child appeared strong and vigorous. Some days afterwards, however, cerebral symptoms began to show themselves, and on the third day from their commencement, coma and death took place. Upon *post-mortem* examination, the pia mater was found considerably injected; the sinuses were gorged with blood of a dark color and syrupy consistence; the choroid plexus was much injected; bloody serum was found in the ventricles; there was effusion of blood at the base of the brain, and the substance of both the brain and cerebellum was of a rosaceous color. The other viscera were healthy, In removing the calvareum, the bone was found of such extreme tenuity as to be quite translucent—it could be depressed by the finger without breaking, and it regained its original form by its own elasticity.

The absence of any violence or protracted duration in the accouchement; the absence of any apparent hereditary predisposition to cerebral disease; and the fact of the mother being well formed, all led him to the conclusion that the death of the child resulted from the thinness and depressibility of the bones of the cranium, allowing of undue pressure on its contents during labor.

The second case was one where the infant became comatose on the second day after birth. Respiration slow, 24 inspirations in the minute; pulse small, and 115. The symptoms became more and more aggravated until the eighth day after their commencement, when death took place, preceded by slight convulsions. At the autopsy there were observed: general congestion of the surface of the brain; serous effusions between the hemispheres; and effusion of blood at the base. The other viscera were healthy. The cranial bones were not so thin in this as in the first case, but the fontanelles were very large, and the sutures open. The diameter of the head was normal.

In the third case, the infant was seized on the sixth day with symptoms of a cerebral nature, and on the eighth a fatal termination ensued. The appearances disclosed upon *post-mortem* examination, were similar to those of the second case, and, as in it, the fontanelles were here of unusual size, and the sutures separated.

It is only necessary to repeat, says M. Charrier, what has been before remarked, that the compression of the skull and brain by the uterus and maternal soft parts during labor, was the cause of the symptoms observed in these cases during life, and the appearances presented upon *post-mortem* examination. The lesions discovered in the three subjects examined, were precisely such as are exhibited by those cases where death results from the application of the forceps, and appear quite sufficient to explain the symptoms manifesting themselves during life.

In a report upon this paper by M. Danyau, and read to the "Société de Chirurgie," it is considered doubtful whether imperfect ossification of the cranial bones can exclusively account for the production of those effects attributed by M. Charrier to this canse. M. Danyau is of the opinion, that other circumstances should have been taken in account, as for example, the condition of the vascular system within the cranium; a predisposition to congestion; liability to rupture of the vessels, etc. In the absence of other indications, however, he considers death, in such cases, to be justifiably assignable to the causes laid down by M. Charrier.

Following up the inquiry on this subject, a communication has since appeared by M. Ancelon, in which he suggests that incomplete ossification of the skull not only conduces directly to pressure on the brain, but from the maternal passages in consequence being imperfectly opened, the liver of the infant suffers by compression, arising indirectly from the same cause. He believes, moreover, that, in those cases of imperfect ossification there generally exists, along with it, some constitutional taint in both the mother and child; and he concludes his paper by remarking:

1st. That owing to some peculiar diathesis, the process of ossification in the fœtus becomes retarded, and the power of resisting pressure during labor consequently diminished.

2d. During labor the maternal soft parts exercise a degree of pressure on the liver, proportionately greater as the ossification of the head is less complete.

3d. The diathesis alluded to, in conjunction with contusion of the liver, explains the fatal result in M. Charrier's cases.—*Monthly Journ. Med.*, June, 1855, from *Gaz. des Hôpitaux*, April 24, and May 5, 1855.

